# Impaired photosynthesis and increased leaf construction costs may induce floral stress during episodes of global warming over macroevolutionary timescales

**DOI:** 10.1038/s41598-018-24459-z

**Published:** 2018-04-18

**Authors:** Matthew Haworth, Claire M. Belcher, Dilek Killi, Rebecca A. Dewhirst, Alessandro Materassi, Antonio Raschi, Mauro Centritto

**Affiliations:** 1 The Italian National Research Council - Tree and Timber Institute (CNR-IVALSA) Via Madonna del Piano 10, Sesto Fiorentino, 50019 Florence, Italy; 2 University of Exeter wildFIRE Lab, Hatherly Labs Prince Wales Road Exeter, EX PS Devon, England; 3 Department of Agrifood Production and Environmental Sciences (DiSPAA), University of Florence Piazzale delle Cascine, 28 50144 Florence, Italy; 4 The Italian National Research Council – Institute of Biometeorology (CNR-IBIMET) Via Giovanni Caproni, 8 50145 Florence, Italy

## Abstract

Global warming events have coincided with turnover of plant species at intervals in Earth history. As mean global temperatures rise, the number, frequency and duration of heat-waves will increase. *Ginkgo biloba* was grown under controlled climatic conditions at two different day/night temperature regimes (25/20 °C and 35/30 °C) to investigate the impact of heat stress. Photosynthetic CO_2_-uptake and electron transport were reduced at the higher temperature, while rates of respiration were greater; suggesting that the carbon balance of the leaves was adversely affected. Stomatal conductance and the potential for evaporative cooling of the leaves was reduced at the higher temperature. Furthermore, the capacity of the leaves to dissipate excess energy was also reduced at 35/30 °C, indicating that photo-protective mechanisms were no longer functioning effectively. Leaf economics were adversely affected by heat stress, exhibiting an increase in leaf mass per area and leaf construction costs. This may be consistent with the selective pressures experienced by fossil Ginkgoales during intervals of global warming such as the Triassic – Jurassic boundary or Early Eocene Climatic Optimum. The physiological and morphological responses of the *G. biloba* leaves were closely interrelated; these relationships may be used to infer the leaf economics and photosynthetic/stress physiology of fossil plants.

## Introduction

Mean global temperatures and the frequency of extreme temperature events have varied throughout Earth history^1–3^. Heat stress may have played a role in the origination, extinction and diversification of plant species during episodes of climatic perturbation^4,5^; yet comparatively little is known about the selective pressures exerted on plants by growth at higher temperatures and the underlying physiological responses. Controlled environment experiments have explored the phenotypic responses of living species with ancient evolutionary origins to reconstruct the likely responses of fossil plants to the atmospheric concentration of carbon dioxide ([CO_2_])^6,7^, the level of oxygen within the atmosphere^8^, fumigation with toxic atmospheric gases^9^, changes in the distribution/quality of incident radiation^10,11^ and a moderate 4 °C increase in temperature^12^. There are comparatively few studies that investigate the impact of heat stress on the physiology and morphology of extant species equivalent to those of fossil plants. In this study, we grew the relict gymnosperm *Ginkgo biloba* ^13^ under controlled environment conditions to examine the impact of heat stress on photosynthetic physiology and leaf morphology.

Fossil Ginkgoales first appear in the Palaeozoic^14^, and are widespread in many Mesozoic^15,16^ and Tertiary^7,17^ sediments. The geographically and temporally extensive fossil record of the Ginkgoales has led to analysis of their potential as indicators of palaeo-environmental and palaeo-climatic conditions. The cuticular micro-morphology of fossil Ginkgoales has been used to reconstruct palaeo-[CO_2_]^7,17–19^, indicate the presence of toxic atmospheric gases^20^, characterise the light environment during leaf development (sun versus shade leaves)^19,21^, estimate photochemical and non-photochemical usage of light energy^22^ and reconstruct leaf economics^23,24^. Analysis of the shape of fossil Ginkgoales has also been used to infer exposure to toxic volcanic gases^9^. The use of fossil Ginkgoales to draw inferences regarding palaeo-climatic conditions, atmospheric composition and plant function has relied upon the analysis of the responses of *G. biloba*, the sole extant Ginkgoales, under experimental and natural conditions to interpret selective pressures faced by fossil plants during global climatic change^7,21,23^. However, to the best of our knowledge, no study to date has investigated the impact of heat stress on *G. biloba*.

Leaf economics provide insights into the physiological characteristics^25^ and environmental growth conditions^26,27^ of a plant. However, it is not possible to directly gauge the leaf economics of fossil plants. The leaf economics of fossil plants can be estimated using relationships between petiole width (PW) and leaf area^28^ and the number of epidermal cells (ED) and leaf density^23^. Few studies of fossil floras have utilised these allometric patterns in leaf macro- and micro-morphology to interpret the physiological responses of fossil plants to fluctuations in palaeo-environmental conditions. One possible explanation may be that the lack of experimental studies quantifying relationships between the energy balance of leaves and the associated physiological and morphological characteristics has limited their application in the reconstruction of the palaeo-physiology of fossil plants.

Episodes of climatic perturbation often accompany extinction events during Earth history^29,30^. The Permian – Triassic^31^, Triassic – Jurassic^4,19,32^, Cretaceous – Tertiary^33,34^, Early Eocene Climatic Optimum^17^ and Paleocene-Eocene Thermal Maximum^5^ are all characterised by increased global temperatures. As mean global temperatures rise, the frequency, duration and severity of heat-waves (transient significant increases in temperature above the mean) also increase^35^. Increased temperature affects the carbon and water use efficiencies of plants^36,37^, and can thus act as a strong selective pressure. Moreover, heat stress incurred during heat-waves can significantly impair photosynthesis^38,39^ and exacerbate the impact of co-occurring stresses such as drought^40^.

As temperatures rise above optimal levels, rates of photosynthesis (*P*_N_) fall due to a decline in the affinity of ribulose-1,5-bisphosphate carboxylase/oxygenase (RubisCO) for CO_2_ reducing carboxylation and increasing oxygenation^36^. The activity of RubisCO is also lower at high temperatures as the function of the enzyme RubisCO activase is reduced^38,41^. Photosystem II (PSII) is strongly affected by high temperatures as the structure and function of the thylakoid membranes, where electron transport occurs within the chloroplast, are highly sensitive to heat stress^39,42^. Impaired function of PSII during heat stress is evident in reductions in the maximum (*F*_v_/*F*_m_) and actual (ΦPSII) quantum efficiencies of electron transport^43^. Nevertheless, plants can develop tolerance to growth at high temperatures through the accumulation of heat shock proteins in the thylakoid membranes^44^. Heat stress can also affect plant metabolism, inducing an increase in levels of respiration in the light (*R*_light_) and dark (*R*_dark_) relative to *P*_N_^27^. Higher temperatures are generally considered to result in greater stomatal conductance (*G*_s_) and increased transpirative water-loss^40^. However, longer exposure to high temperatures may permit adaptation in stomatal behaviour to reduce *G*_s_^27,37^.

Heat stress may play a critical role in driving plant evolutionary responses over geological timescales at intervals of climatic perturbation. For example, the reduction in thermal stress associated with declining [CO_2_] in the Devonian may have enabled the development of large planate leaves^45^, and the reconstruction of leaf architecture of fossil Ginkgoales at the Triassic – Jurassic boundary is consistent with selective pressures induced by heat stress leading to a change in leaf shape to reduce the energy balance of the leaf via lower interception of light^22^. Short-term controlled environment experiments cannot replicate the multi-generational genotypic responses evident in the fossil record associated with natural selection. However, such experiments can provide valuable insights into the acclimatory responses of plants to environmental change and the selective pressures that may be exerted. We exposed the relict gymnosperm *G. biloba* to heat stress to: i) examine the effect on carbon assimilation and photosynthetic light capture; ii) determine the stomatal response of *G. biloba* to higher temperature and potential effects on water use efficiency (WUE), and; iii) gauge the likely effect of heat stress on leaf construction costs to determine whether increased temperatures may have adversely affected leaf economic strategies and thus driven plant evolution during key intervals of climatic change during Earth history using the Triassic – Jurassic boundary as a case study.

## Materials and Methods

### Plant Growth Conditions

Two year-old seedlings of *Ginkgo biloba* were potted into six litre square pots filled with a 5:1 mixture of commercial compost and vermiculite (COMPO Italia, Cesano Maderno, Italy). The *G. biloba* plants were grown from seeds collected from a female tree in Pistoia, Central Italy, which has a warm sub-Mediterranean climate. The seedlings were dormant and leaf development had not yet begun when the plants were placed into two large walk-in growth rooms in February 2015. Five plants were placed in each growth room. The plants were watered to pot capacity every two days and once a week were provided with a commercial liquid plant fertiliser (COMPO Concime Universale, NPK 7-5-7, B, Cu, Fe, Mn, Mo, Zn) to facilitate nutrient availability at free access rates. The growth chambers maintained conditions of 400 ppm [CO_2_], relative humidity of 60% and 16 hours of daylight (14 hours at full photosynthetically active radiation, PAR, levels of 1000 μmol m^−2^ s^−1^ with two one-hour periods of simulated dawn/dusk where light intensity was incrementally increased/decreased – details of the light spectrum are provided in Supplementary Information). One chamber operated a day/night time temperature regime of 25/20 °C (hereafter referred to as the 25 °C treatment) and the second chamber operated a day/night temperature of 35/30 °C (hereafter referred to as the 35 °C treatment). Changes in temperature followed those of PAR, with a one-hour ramping period at dawn/dusk. To avoid any potential chamber effects the growth rooms were alternated every two weeks – no significant differences were observed in gas exchange measurements conducted under the same conditions in different growth chambers. The plants were grown for three months under controlled environment conditions to allow full leaf development before physiological measurements were performed over a two week period after the 12^th^ week.

### Leaf gas exchange, chlorophyll fluorescence and chlorophyll content analysis

A PP-Systems Ciras-2 attached to a 2 cm^2^ PLC6(U) leaf cuvette and LED light unit (PP-Systems, Amesbury, Massachusetts, USA) was used to analyse leaf gas exchange. A minimum of five leaves were analysed per plant with five plants for each temperature treatment. Point measurements of *P*_N_ and *G*_s_ were performed using cuvette conditions identical to the growth conditions of both treatments: a temperature of 25 or 35 °C, PAR of 1000 μmol m^−2^ s^−1^ and [CO_2_] of 400 ppm. The instantaneous transpiration efficiency was calculated as the ratio of *P*_N_ to *G*_s_. The maximum rate of *P*_N_ (*P*_N max_) of leaves from both treatments was measured using cuvette conditions of 25 °C, 2000 μmol m^−2^ s^−1^ PAR and 2000 ppm [CO_2_]. The Kok^46^ method was used to estimate *R*_light_ by decreasing PAR at low levels of intensity (400, 300, 200, 150, 100, 75, 50, 30, 20 and 10 μmol m^−2^ s^−1^). Respiration in the dark was measured by switching off the LED light unit after the Kok protocol, shading the plant and recording the rate of CO_2_ efflux from the leaf after values had remained stable for 5–10 minutes. Both *R*_light_ and *R*_dark_ were determined at the growth temperature of the plants in the controlled environment chambers. The effect of variation in *C*_i_ on *R*_d_ was corrected using the iterative method of Kirschbaum and Farquhar^47^. The maximum (*F*_v_/*F*_m_) and actual (ΦPSII) quantum efficiencies of PSII and non-photochemical quenching (NPQ) values of each leaf were recorded using a FMS-2 modulated fluorimeter (Hansatech, Norfolk, King’s Lynn, UK) (saturating pulse of 10,000 μmol m^−2^ s^−2^) and dark adaptation clips after 30 minutes of dark adaptation and exposure to actinic light of 1000 μmol m^−2^ s^−1^ for a minimum of 10 minutes after the first saturating pulse^48,49^. Chlorophyll content of each leaf was estimated from the average of five readings per leaf using a Konica Minolta SPAD-502 (Konica Minolta, Tokyo, Japan) and the calibration of Marenco, *et al*.^50^.

### Leaf economics and construction costs

After physiological measurements, leaves were removed from the plants, the petiole cut from the leaf and the leaf photographed using a Sony DSC-T99 14 megapixel camera. Leaf area was calculated from digital images using ImageJ (National Institutes of Health, Bethesda, Maryland, USA). The fresh petiole length and width were recorded using digital callipers with a precision of 0.01 mm (Draper Tools, Hampshire, UK). After digital images were taken of the leaves, dental impression gel was applied to the adaxial leaf surface for 30 minutes. The dental impression gel was then removed and the leaves were dried for four days at 70 °C. After the mass of individual leaves remained stable for a minimum of two days, indicating that the leaves were dried thoroughly, their weight was recorded. The leaf mass per area (LMA) of each leaf was calculated as the mass of the leaf (in grams) divided by its area (expressed as m^−2^).

After drying, each leaf was cut into two approximately equal halves. The half of the leaf designated for elemental analysis was ground in liquid nitrogen using a pestle and mortar and then the carbon and nitrogen content was determined using a Carlo Erba NA 1500 CHNS Analyzer (Carlo Erba, Milan, Italy). The remaining halves of each leaf were used for micro-calorimetry using a Federal Aviation Adminstration microcalorimeter (Fire Testing Technology, East Grinstead, UK). The maximum temperature (*T*_max_: the temperature at which the maximum rate of decomposition of virgin fuel is reached), peak heat release (pHRR: the most intense flux of heat during the combustion of the leaf material, this indicates the maximum decomposition rate of the leaves which is related to the volatile gas flux of the material), heat capacity (HRCap: the maximum capability of the leaf material to release combustion heat per degree of temperature during pyrolysis; this measure provides an indication of the resistance of the leaves to thermal degradation) and total heat release (THR: the total energy released by the leaf during combustion) was determined for each leaf on a g^−1^ dry mass basis. The THR during combustion and the amount of carbon and nitrogen within the leaves were used to estimate leaf construction costs using the method of Williams, *et al*.^51^, termed CC_w_ (equation 1):

1 $${\rm{CCw}}=\frac{(0.06968{{\rm{\Delta }}{\rm{H}}}_{{\rm{c}}}-0.065)(1-{\rm{A}})+0.5359{k}{\rm{N}}}{{{\rm{E}}}_{{\rm{G}}}}$$

Where, CC_w_ is the energetic cost of tissue construction (g glu g^−1^ dry mass); ∆H_c_ is the ash free heat of combustion (KJ g^−1^); A is the ash content after combustion; *k* is the oxidation state of nitrate, a value of 5 was used following Wullschleger, *et al*.^52^; N is the nitrogen content (g g^−1^ dry mass), and; E_G_ is the efficiency of conversion of dry matter to heat, a value of 0.89 was used following Williams, *et al*.^51^. The method of de Vries, *et al*.^53^ (CC_v_) that does not incorporate analysis of the energy released during combustion was also used (equation 2):

2 $${{\rm{CC}}}_{{\rm{v}}}=(5.39{\rm{C}}+0.8{\rm{A}}+5.64{{f}}_{{\rm{N}},{\rm{h}}}{\rm{N}}-1.191)(1+{{\rm{r}}}_{{\rm{T}}})$$

Where, C is the carbon content (g g^−1^ dry mass); *f*_N,h_ is the fraction of nitrogen assimilated heterotrophically, a value of 0.5 was used following Wullschleger, *et al*.^52^, and; r_T_ is the cost of translocating photosynthates, a value of 5.3% was used^54^.

### Volatile compound and lignin analysis

Individual dried leaves were ground in liquid nitrogen using a pestle and mortar. Volatile compounds were extracted by sonication in *n*-hexane, with butylated hydroxytoluene (BHT) added as an internal standard. The hexane extract was analysed by GC-MS, using an Agilent 7200 series accurate mass Q-TOF GC-MS together with a 7890 A GC system (Agilent Technologies, Santa Clara, USA), equipped with an EI (electron ionisation) ion source. Then 5 μl of each sample was injected into a non-deactivated, baffled glass liner with a 12:1 split ratio (14.448 ml min^−1^ split flow) and the inlet temperature was maintained at 250 °C. A Zebron semi-volatiles (Phenomenex, Torrance, USA) column (30 m × 250 μm × 0.25 μm) coupled with a 10 m guard column, was maintained at a constant helium flow of 1.2 ml min^−1^. The oven temperature was initially 70 °C increasing to 310 °C at a rate of 15 °C min^−1^. It then remained constant at 310 °C for 6 minutes. The MS emission current and emission voltage were held at 35 μA and 70 eV respectively. The mass range was set from 50 to 600 amu, with an acquisition rate of 5 spectra s^−1^.

Lignin was analysed using the acetyl bromide method^55^. The ground leaves were washed to produce a protein-free cell wall preparation. Samples (2 mg) of the cell wall preparations were incubated in acetyl bromide (0.5 ml, 25% v/v in acetic acid) at 50 °C for two hours. After incubation, sodium hydroxide (0.9 ml, 2 M) and hydroxylamine hydrochloride (0.1 ml, 1 M) were added, along with acetic acid (5 ml). The lignin content was quantified by measuring the absorbance at 280 nm using a UV-1600PC Spectrophotometer (VWR, Leicestershire, UK).

### Leaf micro-morphological analysis

Impressions of the adaxial surface of the *G. biloba* leaves were taken using dental impression gel (Coltène President Light Body Material, Cuyahoga Falls, Ohio, USA). These were then used to create nail varnish ‘positives’ that were mounted onto glass slides^56^ and imaged using a Leica DM2500 microscope attached to a Leica DFC300FX camera (Leica Microsystems, Wetzlar, Germany). Epidermal cell density (ED) counts were consistently performed on cells in the outer lobe of the *G. biloba* leaf to avoid concentrations of veins and/or excessively densely packed epidermal cells at the base of the leaf^23^. A 0.16 mm^2^ grid (0.4 × 0.4 mm) was applied to digital images of the cuticle and ED of 10 images recorded for each leaf. Rarefraction analysis of the ED counts for extant *G. biloba* indicated that mean ED values stabilised after three to five images were counted.

### Statistical analyses

Statistical analyses were performed using SPSS 20 (IBM, New York, USA). A one-way ANOVA was used to assess differences in variance between temperature treatments. Linear regression was used to assess possible relationships between leaf morphology and physiology.

### Data availability statement

All data generated or analysed during this study are included in this published article.

## Results

**Figure 1:**
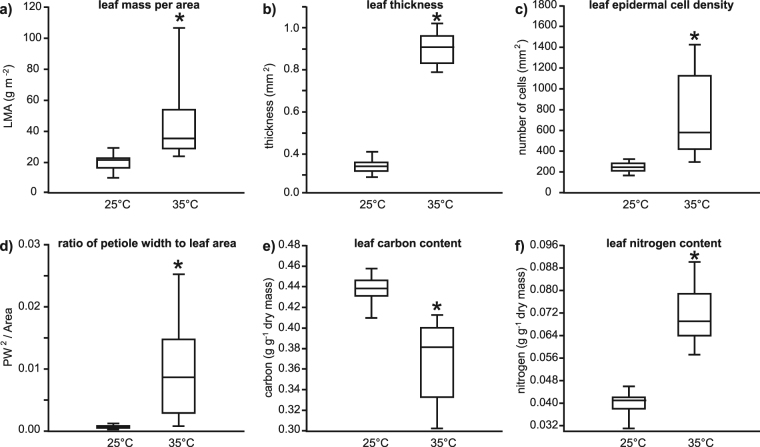
Box plots representing the effects of growth at 25 and 35 °C on the morphology of *Ginkgo biloba* leaves: (**a**) leaf mass per area (one-way ANOVA F_1,53_ = 41.5; *P* = 3.5 × 10^−8^); (**b**) leaf thickness (one-way ANOVA F_1,53_ = 1502.7; *P* = 1.4 × 10^−40^); (**c**) leaf epidermal cell density (one-way ANOVA F_1,53_ = 53.3; *P* = 1.5 × 10^−9^); (**d**) ratio of petiole width (PW) to leaf area (one-way ANOVA F_1,53_ = 52.2; *P* = 2.0 × 10^−9^); (**e**) leaf carbon content (one-way ANOVA F_1,53_ = 96.1; *P* = 1.6 × 10^−13^), and; (**f**) leaf nitrogen content (one-way ANOVA F_1,53_ = 316.2; *P* = 5.4 × 10^−4^). The box signifies the distribution of the 25–75% quartiles, the median is represented by a horizontal line within the box, horizontal bars either side of the box indicate minimum/maximum values. *indicates significant different between the 25 and 35 °C treatments at the 0.05 significance level.

**Figure 2:**
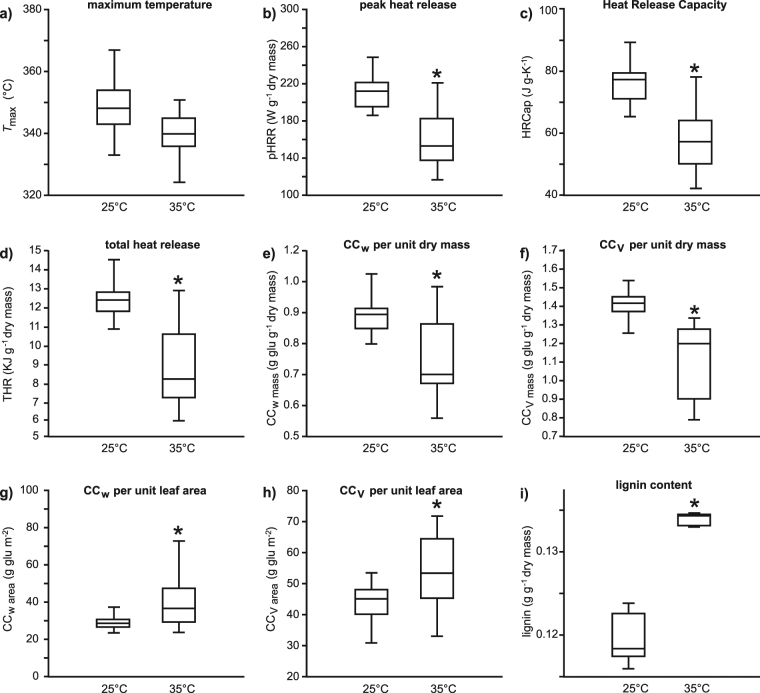
Box plots representing the effects of growth at 25 and 35 °C on the parameters determined by calorimetry and construction costs of *Ginkgo biloba* leaves: (**a**) maximum heat release (one-way ANOVA F_1,53_ = 3.801; *P* = 0.057); (**b**) peak heat release (pHRR)(one-way ANOVA F_1,53_ = 58.9; *P* = 9.0 × 10^−10^); (**c**) heat release capacity (HRCap)(one-way ANOVA F_1,53_ = 61.9; *P* = 4.6 × 10^−10^); (**d**) total heat release (THR)(one-way ANOVA F_1,53_ = 90.7; *P* = 4.5 × 10^−13^); (**e**) leaf construction cost per unit dry mass following Williams, *et al*.^51^ (CC_w mass_) (one-way ANOVA F_1,53_ = 34.2; *P* = 3.1 × 10^−7^); (**f**) leaf construction cost per unit dry mass following de Vries, *et al*.^53^ (CC_v mass_) (one-way ANOVA F_1,53_ = 65.2; *P* = 8.4 × 10^−11^); (**g**) CC_w_ per unit leaf area (CC_w area_) (one-way ANOVA F_1,53_ = 14.5; *P* = 3.6 × 10^−4^); (**h**) CC_v_ per unit leaf area (CC_v area_) (one-way ANOVA F_1,53_ = 10.6; *P* = 0.00195), and; (**i**) lignin content per unit dry mass (one-way ANOVA F_1,53_ = 114.9; *P* = 8.4 × 10^−7^). Box plots presented as in Fig. 1.

**Figure 3:**
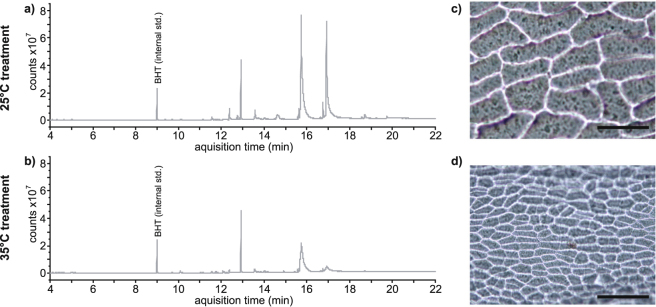
Example GCMS spectra of lignin and volatile compound content of *Ginkgo biloba* leaves and images of impressions of the adaxial epidermis grown in the 25 °C treatment (**a**,**c**) and 35 °C treatment (**b**,**d**). Scale bar on images of epidermal micromorphology indicates 100 μm.

**Figure 4:**
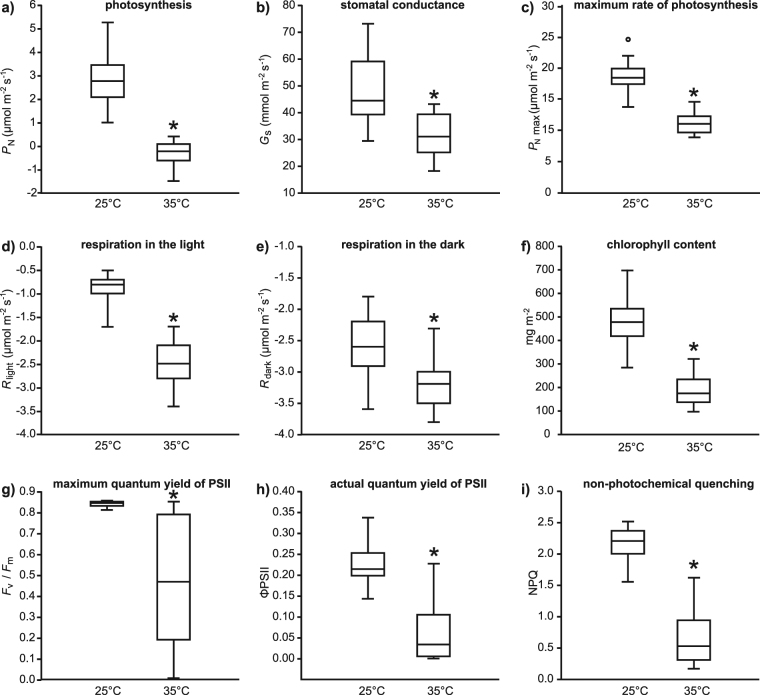
Box plots representing the effects of growth at 25 and 35 °C on the physiology of *Ginkgo biloba* leaves: (**a**) photosynthesis (one-way ANOVA F_1,53_ = 157.6; *P* = 1.7 × 10^−17^); (**b**) stomatal conductance (one-way ANOVA F_1,53_ = 31.3; *P* = 7.9 × 10^−7^); (**c**) maximum rate of photosynthesis (one-way ANOVA F_1,53_ = 181.2; *P* = 9.8 × 10^−19^); (**d**) respiration in the light (one-way ANOVA F_1,53_ = 261.1; *P* = 4.0 × 10^−22^); (**e**) respiration in the dark (one-way ANOVA F_1,53_ = 30.1; *P* = 1.2 × 10^−6^); (**f**) foliar content of chlorophyll a and b (one-way ANOVA F_1,53_ = 150.1; *P* = 4.3 × 10^−17^); (**g**) maximum quantum yield of PSII (one-way ANOVA F_1,53_ = 52.6; *P* = 1.8 × 10^−9^); (**h**) actual quantum yield of PSII (one-way ANOVA F_1,53_ = 106.08; *P* = 3.0 × 10^−14^), and; (**i**) non-photochemical quenching (one-way ANOVA F_1,53_ = 250.4; *P* = 1.0 × 10^−23^). Box plots presented as in Fig. 1.

Growth at 35 °C strongly influenced the morphology (Fig. 1), composition (Figs 2 and 3) and physiology (Fig. 4) of the leaves of *G. biloba*. Leaf mass per area was significantly increased by 58% in leaves developed at the higher temperature (Fig. 1a). This higher LMA was associated with increased leaf thickness (Fig. 1b) and ED (Figs 1c, 3c,d). The ratio of PW^2^ to leaf area was also significantly greater in leaves from the 35 °C treatment (Fig. 1d). Higher growth temperature resulted in 15% lower leaf carbon content (Fig. 1e), and a corresponding 79% increase in the amount of nitrogen (Fig. 1f) when measured on the basis of leaf dry weight. Microcalorimetry revealed a ~10 °C difference in the temperature at which the maximum decomposition of the leaf occurred (Fig. 2a), indicating that the compounds in the leaves grown at 35 °C were more easily degradable than those grown in 25 °C. The pHRR (Fig. 2b), HRCap (Fig. 2c) and THR (Fig. 2d) per unit dry mass were significantly lower in leaves from the 35 °C treatment. The greater THR in leaves developed at 25 °C, contributed towards 17% lower construction costs per unit dry mass in leaves from the 35 °C treatment (Fig. 2e,f). However, when construction costs were calculated on a leaf area basis, leaves that developed in the 35 °C treatment were on average 36% more expensive than their counterparts from the 25 °C treatment (Fig. 2g,h). This also appears to be reflected in the chemical analyses, where the leaves grown at 35 °C had significantly lower lignin content than the leaves grown at 25 °C (Fig. 2i). The leaves grown at 35 °C also generated significantly less volatile waxy compounds than those grown at the lower temperature (Fig. 3).

Photosynthesis (Fig. 4a), *G*_s_ (Fig. 4b) and *P*_N max_ (Fig. 4c) were all lower in leaves from the 35 °C treatment. In contrast, rates of respiration in the light (Fig. 4d) and dark (Fig. 4e) were larger in leaves from the higher temperature (Fig. 4d,e), indicating that the lower rates of *P*_N_ at 35 °C were not accompanied by reduced metabolic activity. Lower *G*_s_ and transpirative water-loss at 35 °C did not translate into improved transpiration efficiency due to diminished *P*_N_ (*P*_N_/*G*_s_: 25 °C treatment = 57.7 ± 3.0 μmol CO_2_ mol^−1^ H_2_O; 35 °C treatment = −10.1 ± 3.0 μmol CO_2_ mol^−1^ H_2_O). The amount of chlorophyll per unit leaf area was 61% lower in leaves from the 35 °C treatment (Fig. 4f), and this corresponded to a 72% reduction in ΦPSII (Fig. 4h). Non-photochemical quenching of *G. biloba* leaves was reduced by 69% on average in the 35 °C treatment (Fig. 4i).

**Figure 5:**
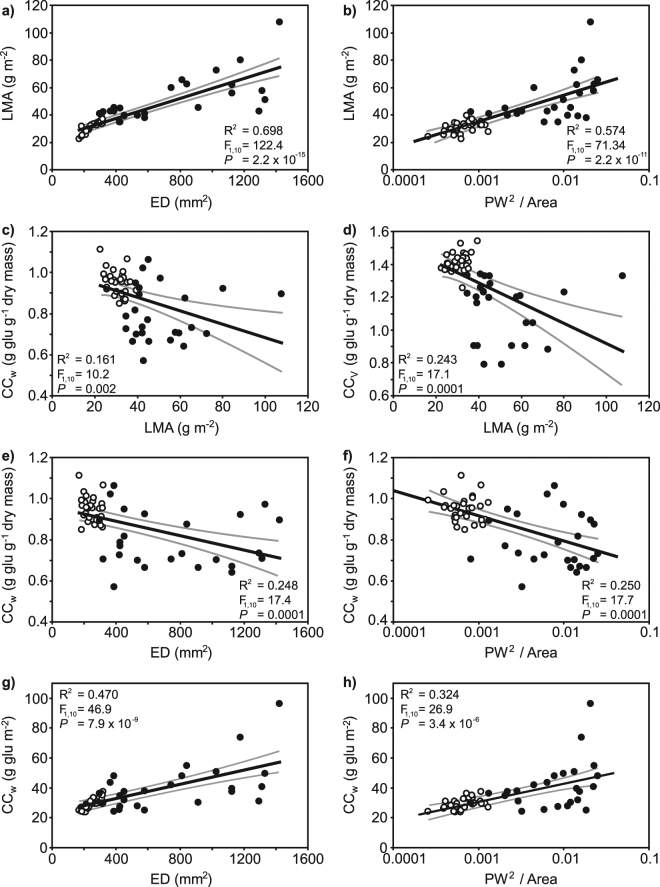
Correlations between leaf macro- and micro-morphology with leaf economic and construction cost parameters of *Ginkgo biloba* leaves developed in 25 °C (white fill symbols) and 35 °C treatments (black fill symbols): (**a**) relationship between LMA and epidermal cell density (ED); (**b**) relationship between LMA and the ratio of PW^2^ (where PW = petiole width) to leaf area plotted on a logarithmic scale; (**c**) relationship between CC_w_ per unit dry mass and LMA; (**d**) relationship between CC_v_ per unit dry mass and LMA; (**e**) relationship between CC_w_ per unit dry mass and ED; (**f**) relationship between CC_w_ per unit dry mass and the ratio of PW^2^ to leaf area plotted on a logarithmic scale; (**g**) relationship between CC_w_ per unit leaf area and ED, and; (**h**) relationship between CC_w_ per unit leaf area and the ratio of PW^2^ to leaf area plotted on a logarithmic scale. R^2^, F and *P* values indicate the results of linear regression. The central black line indicates the line of best fit. The grey lines either side of the best-fit line indicate 95% confidence intervals of the mean.

**Figure 6:**
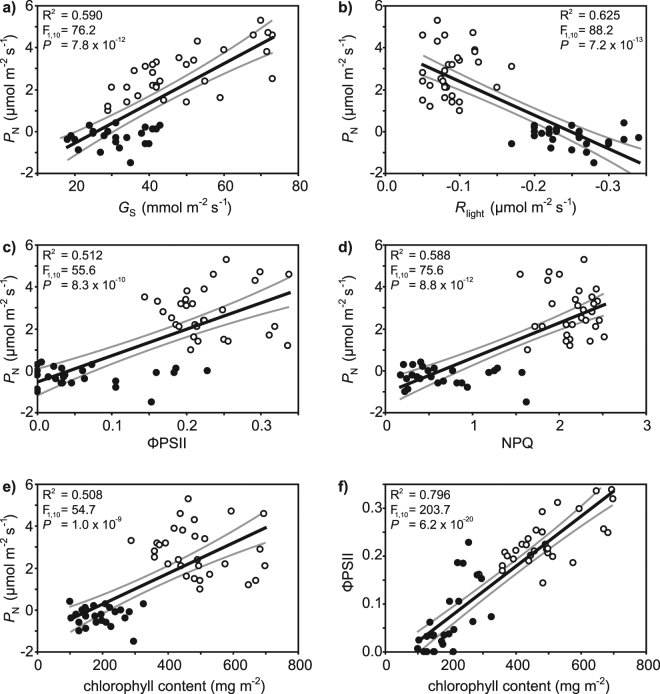
The effect of growth at 25 °C (white fill symbols) and 35 °C (black fill symbols) on correlations between physiological parameters in leaves of *Ginkgo biloba*: (**a**) relationship between *P*_N_ and *G*_s_; (**b**) relationship between *P*_N_ and *R*_light_; (**c**) relationship between *P*_N_ and ΦPSII; (**d**) relationship between *P*_N_ and NPQ; (**e**) relationship between *P*_N_ and foliar chlorophyll content, and; (**f**) relationship between ΦPSII and foliar chlorophyll content. R^2^, F and *P* values indicate the results of linear regression. The central black line indicates the line of best-fit. The grey lines either side of the best fit line indicate 95% confidence intervals of the mean.

The LMA of the *G. biloba* leaves exhibited significant positive correlations with ED (Fig. 5a) and the ratio of PW^2^ to leaf area (Fig. 4b). Less strong negative relationships were observed between construction cost per unit dry mass with LMA (Fig. 5c,d), ED (Fig. 5e) or the ratio of PW^2^ to leaf area (Fig. 5f). However, when leaf construction costs were calculated per unit leaf area, slightly more robust positive relationships were observed with ED (Fig. 5g) and the ratio of PW^2^ to leaf area (Fig. 5h). Relationships between photosynthetic and protective physiology of the *G. biloba* leaves were also strongly affected by temperature (Fig. 6). A strong positive relationship was observed between *P*_N_ and *G*_s_ (Fig. 6a), but *P*_N_ was negatively associated with *R*_light_ (Fig. 6b). Photosynthesis measured using gas exchange was positively related to ΦPSII (Fig. 6c) and NPQ (Fig. 6d) measured via chlorophyll fluorescence. The concentration of chlorophyll within the *G. biloba* leaves was also positively related to CO_2_ assimilation determined using gas exchange (Fig. 6e) and chlorophyll fluorescence (Fig. 6f) techniques.

**Figure 7:**
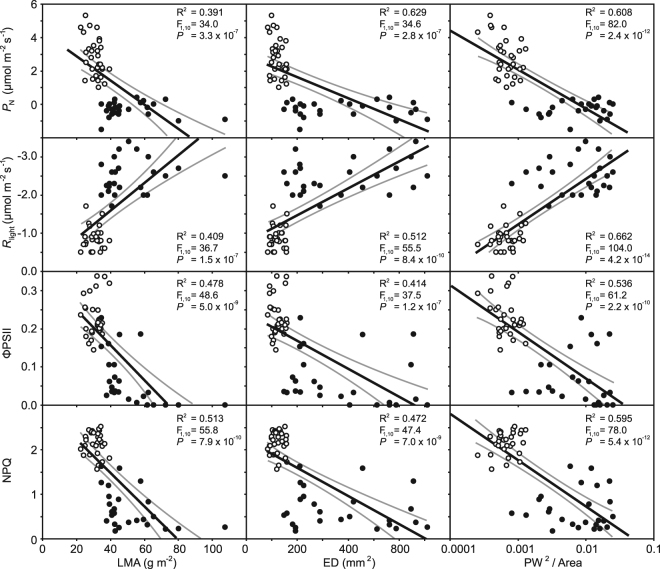
Correlations between photosynthesis, *R*_light_, ΦPSII and NPQ and morphological characteristics of leaves of *Ginkgo biloba* grown at 25 °C (white fill symbols) and 35 °C (black fill symbols). The ratio of PW^2^ to area is plotted on a logarithmic scale. R^2^, F and *P* values indicate the results of linear regression. The central black line indicates the line of best fit. The grey lines either side of the best-fit line indicate 95% confidence intervals of the mean.

The pronounced impact of temperature on the leaves of *G. biloba* was also apparent in relationships of photosynthetic physiology to LMA and the two possible proxy methods for estimating the LMA of fossil plants (Fig. 7). Photosynthesis, ΦPSII and NPQ were negatively related to LMA. Indeed, the relationship of *P*_N_ with ED or ratio of PW^2^ to leaf area was stronger than that with LMA. Respiration in the light exhibited negative relationships to LMA, ED and PW^2^ to leaf area. These correlations may suggest that the macro- and micro-morphology of *G. biloba* leaves may provide a basis to infer the status of photosynthetic and protective physiology within the leaf.

## Discussion

This study has shown that heat stress impaired *P*_N_ and affected leaf morphology in *G. biloba*. The natural habitat of *G. biloba* is not well defined as the species was restricted to refugia during Pleistocene glacial episodes and has experienced centuries of managed planting^57^. Nonetheless, *G. biloba* has been cultivated extensively over a wide-range of environments with differing climates^58^, and grows successfully in warm humid climates with mean summer temperatures of 30 °C where midday temperatures can exceed 40 °C^23^. Indeed, the female tree which was the source of the seeds and the nursery where the seedlings grew were located in a region with a warm sub-Mediterranean climate, suggesting that adaptation to cooler temperatures was not associated with the pronounced response to heat stress observed in the study.- Exposure to 35 °C for 14 hours each day may have exceeded the tolerance of *G. biloba* by progressively degrading the protective physiology. As the use of energy for photochemistry declines, an increase in the dissipation of energy as heat is frequently observed^59^. However, *G. biloba* grown in the 35 °C treatment showed lower levels of NPQ than those grown at 25 °C, suggesting that this protective mechanism was not functioning as effectively at the higher temperature (Fig. 4i). The pronounced reduction in ΦPSII at 35 °C is consistent with previous studies suggesting that the structure and function of the thylakoid membrane is particularly vulnerable to heat stress^39,42,43^. The close correlation between ΦPSII and chlorophyll content indicates that the increased pigment content in leaves from the 25 °C treatment enabled greater light harvesting associated with an increased availability of PSII reaction centres for electron transport involved in CO_2_ fixation^60^. The lower levels of photosynthetic CO_2_ assimilation observed in *G. biloba* (Fig. 4a) may be due to decreased affinity for CO_2_ relative to O_2_ resulting in an increase in photorespiration relative to *P*_N_, reduced solubility of CO_2_^36,61^ or reduced activity of RubisCO activase^39,62^. This reduced carboxylation would decrease the capacity of photosystem I to accept electrons from PSII^63^, further exacerbating the deleterious effects of excess energy on the thylakoid membranes.

Higher temperatures negatively affected the carbon balance of the *G. biloba* leaves via reduced *P*_N_ (Fig. 4a) and increased respiration (Fig. 4d,e), this is consistent with the decrease in lignin and volatile waxy compound production in the leaves grown at 35 °C (Figs 2i and 3). In contrast to previous studies where a short-term increase in temperature induced an increase in *G*_s_ and transpirative water-loss^40,64^, a reduction in *G*_s_ at the higher temperature was observed (Fig. 4b). This stomatal acclimation is consistent with *Populus nigra* grown for eight weeks at 35 °C^37^. Despite the reduced transpirative water-loss at 35 °C, this did not translate into improved transpiration efficiency due to impaired CO_2_ assimilation. Moreover, the lower transpirative cooling^65^ would exacerbate the impact of growth at the higher temperature on the photosynthetic apparatus. As most plants experience heat stress over a few hours^66^, the prolonged exposure to higher temperature as part of a simulated heat-wave in this study likely induced significant damage to PSII and the protective xanthrophyll cycle (Fig. 4i). This damage to PSII was likely caused by the decline in the amount of energy utilised in photochemistry due to reduced assimilation of CO_2_ and lower capacity for transpirative cooling associated with stomatal adaptation to the higher temperature.

The chemical analyses of leaf composition, elemental analysis of whole leaf carbon and nitrogen content and the microcalorimetry analyses provide evidence of changes in leaf composition between the two treatments. The decrease in carbon content, lignin and volatile waxy compounds in the 35 °C treatment *G. biloba* leaves highlights that investment in the structure of the leaves is altered at the higher temperature. Specifically, fatty acids and volatile waxes appear to be more limited in the leaves grown at the higher temperature. This is further reflected in the energy content responses measured by the microcalorimeter. The leaves from the 35 °C treatment were found to reach maximum decomposition rate at lower temperatures when heated at the same ramp rate as leaves grown in the 25 °C treatment. This indicates that the leaves grown at 35 °C contain compounds that are easier to break-down, implying that their investment is likely to be in short-lived cellulosic compounds rather than longer-lived lignin (eg. Fig. 2i). There is also a large difference in pHRR between the two sets of leaves, with the leaves grown at 25 °C releasing 60 W g^−1^ more energy than those grown at the higher temperature. This is likely related to the significantly lower abundance of volatile waxy compounds in the higher temperature leaves. The same is observed for HRCap and THR, which both indicate that the structural investment and the complexity of compounds is lower in the high temperature leaves; implying that growth conditions have a significant influence on the energy content of the leaves. Therefore, the damage induced to the photosynthetic and light harvesting apparatus appears to have a large influence on investment in the construction of leaves. Not only are the leaves more costly to produce, but the plants appeared to have more limited resources to invest in longer chained carbon-based compounds.

Analyses of leaf economics has been suggested to provide insights into the physiological and morphological adaptations of plants to their environment^67^. However, the application of leaf economics to gauge the palaeo-ecology and physiology of fossil plants has been relatively limited. It is not possible to directly determine the leaf economics of a fossil plant or its physiological status. Instead, allometric relationships in leaf area and PW^28^ and the density of epidermal cells^23^ have been used to estimate the LMA of fossil plants. The results of our growth experiments and analysis suggest that patterns in leaf morphology correlate closely with physiological parameters such as *P*_N_, *R*_light_ and ΦPSII (Fig. 7) as well as structural investment (Fig. 2) and therefore may provide novel palaeo-physiological data from observations of leaf fossils. To assess this possibility we have considered changes in Ginkgoales leaf morphology across the Triassic-Jurassic boundary, global warming event as preserved at locations in East Greenland^68^.

**Figure 8:**
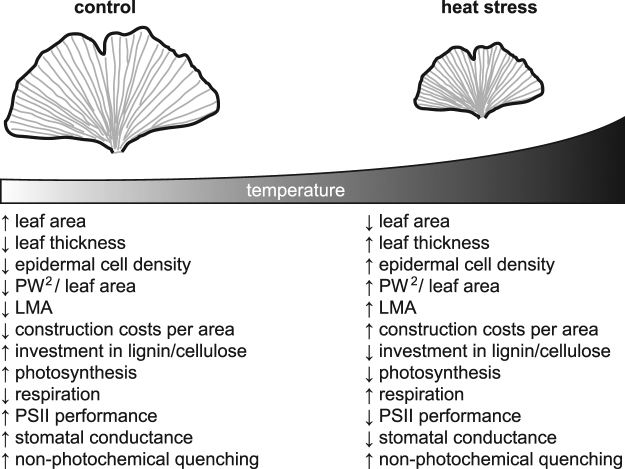
A summary of the physiological and morphological effects of heat stress on *Ginkgo biloba* leaves. Arrows facing up and down indicate respectively higher and lower values of each parameter.

At the Triassic – Jurassic boundary mean global temperatures are proposed to have risen by 2.5 to 5.0 °C due to increased palaeo-atmospheric [CO_2_]^4,19,69^, with much wider regional variations of more than 10 °C in temperature^70^. Alongside the increased incidence of heat-waves^35^, this would have adversely affected CO_2_-uptake in Late Triassic Ginkgoales. Moreover, the leaf mass per area of fossil Ginkgoales is considered to have increased by 40–60% towards the End Triassic^23,24^, and leaf architecture adjusted to reduce energy interception^22^. Heat stress induced a 57.7% increase in the LMA of *G. biloba* (Fig. 1a), and a rise in [CO_2_] from 380 to 1500 ppm has been shown to increase the LMA of *G. biloba* by 30.6%^8^. The results of these observations under controlled environment conditions would suggest that a rise in LMA of fossil Ginkgoales at the end of the Triassic was likely due to higher [CO_2_] and temperature. Nevertheless, it is unclear whether a combination of heat stress and extremely elevated [CO_2_] would have a cumulative, synergistic or antagonistic impact on the leaf economics of *G. biloba*. The controlled environment analysis conducted in this study indicates that rising temperatures at the Triassic – Jurassic boundary would also have increased the construction cost of foliage per unit area of the leaf (Fig. 2). In essence, this equates to greater cost accompanied by diminished returns in the form of reduced interception of PAR for *P*_N_ and less favourable rates of *P*_N_ to photorespiration. This shift in growth conditions as temperatures rose would make leaves of Ginkgoales more expensive and with lower photosynthetic returns; possibly accounting for regional extinctions of Ginkgoales at the Triassic – Jurassic boundary^71^ (for a summary of effects see Fig. 8). The *T*_max_ (Fig. 2a), HRCap (Fig. 2c), lignin content (Fig. 2i) and the abundance of volatile waxy compounds data indicate a lower structural investment and a lower energy content of the leaves from the higher temperature. A similar effect of higher temperatures on leaf development may have affected the combustion characteristics^3,72^ and preservation potential^8^ of fossil plant material during the transition from the Triassic to Jurassic.

Heat stress induced a significant reduction in the capacity for photosynthetic electron transport (Fig. 4g,h) and CO_2_ uptake (Fig. 4a,c). This lower *P*_N_ resulted in lower transpiration efficiency of *G. biloba* in the 35 °C treatment. If such a scenario were replicated at episodes of global warming during Earth history this would reduce the photosynthetic performance of C3 species; in particular, those individuals with lower transpiration efficiencies, or exposed to high evapotranspirative demand. Some C3 species may have adapted to higher temperatures via increased levels of RubisCO, thus countering the impact of reduced RubisCO carboxylation^63^. However the formation of RubisCO requires nitrogen, and as nitrogen is limiting in many terrestrial environments^73,74^, an increase in RubisCO may not be feasible for many plants faced with heat stress. The degradation of the protective mechanisms involved in the maintenance and stabilisation of the thylakoid membranes during heat stress^44,59^ would also contribute to plant stress at the Late Triassic^22^. This would be compounded by reduced *G*_s_ at high temperatures (Fig. 4b), resulting in reduced transpirative cooling^75^. The pattern of leaf economic responses to heat stress observed under controlled climate conditions are consistent with the micro^23^- and macro-morphological^8,9^ patterns of fossil Ginkgoales at the Triassic – Jurassic boundary, suggesting that heat stress may have potentially played a role in floral turnover recorded at the boundary^71,76^. Estimates of the leaf economics of fossil Ginkgoales based upon epidermal micromorphology suggest that LMA values increased by 40.1 (from 71.4 ± 2.0 to 100.0 ± 3.5 g m^−2^ ^24^) to 54.1% (from 85.5 ± 2.6 to 132.2 ± 4.1 g m^−2^ ^23^) at the Triassic – Jurassic boundary. The reconstructed LMA values of the fossil Ginkgoales are comparable to those observed in the 35 °C treatment (Fig. 1a) and *G. biloba* trees growing in a warm humid sub-Mediterranean climate^23^. Higher temperatures, especially those significantly above normal levels associated with heat-waves, may act as a strong selective pressure in the extinction, origination and diversification of plant species over geological timescales.

Our fossil analysis suggests that the correlations we report between metabolism and photosynthesis with LMA, lignin content, volatile compounds, ED and the ratio of PW^2^ to leaf area may allow inferences to be drawn regarding the physiological status of fossil plants; particularly in the context of a stratigraphic sequence where the relative changes in leaf morphology through the sequence provide context to the physiological responses and the selective pressures (such as variations in palaeo-[CO_2_] or -temperature) that may have driven these changes.

Higher temperatures induced a significant increase in the LMA of *G. biloba* leaves (Fig. 1a), consistent with observations of higher LMA in *G. biloba* from warmer areas^22^ and controlled environment temperature experiments involving other species^27^. Heat stress also resulted in the allometric relationships between LMA and both ED (Fig. 5a) and PW^2^ to leaf area (Fig. 5b) becoming more variable. This may suggest that stress conditions were adversely affecting leaf development^77^, which is also reflected in the shift to higher nitrogen contents and lower lignin and volatile compound investment. Nonetheless, both ED and PW^2^ to leaf area showed significant relationships to LMA that could be utilised in the reconstruction of leaf economics in fossil Ginkgoales during episodes of temperature change for example the Early Eocene Climatic Optimum^17^. The leaves of *G. biloba* can either ‘short’ or ‘long’ petiole morphotypes^78^. All of the leaves analysed in this study were of the short petiole morphotype. The scaling relationship between PW^2^ to leaf area^28^ becomes less robust in *G. biloba* when a mixture of short and long petiole leaves are analysed^78^. Furthermore, the PW^2^/leaf area approach relies upon a high degree of preservation where the entire leaf area is intact with the petiole still attached; however, many fossil leaves are fragmented during transport prior to deposition eg. Oldham^79^, possibly limiting the application of this method. Nevertheless, *G. biloba* modifies the physiology and morphology of its leaves in response to heat stress. The photosynthetic apparatus, leaf morphology and leaf composition are clearly affected by environmental conditions; moreover, they are intrinsically linked during leaf development. As such, the interrelated correlations between leaf morphology, construction costs and physiology can be used to reconstruct the likely responses of fossil plants to environmental change, and infer the selective pressures that have shaped plant evolution during episodes of global warming.

## Electronic supplementary material

Supplementary Information
