# Publisher Correction: Impaired photosynthesis and increased leaf construction costs may induce floral stress during episodes of global warming over macroevolutionary timescales

**DOI:** 10.1038/s41598-018-26883-7

**Published:** 2018-05-31

**Authors:** Matthew Haworth, Claire M. Belcher, Dilek Killi, Rebecca A. Dewhirst, Alessandro Materassi, Antonio Raschi, Mauro Centritto

**Affiliations:** 1 The Italian National Research Council - Tree and Timber Institute (CNR-IVALSA), Via Madonna del Piano 10, Sesto Fiorentino, 50019 Florence, Italy; 2 University of Exeter wildFIRE Lab, Hatherly Labs Prince Wales Road Exeter, EX PS, Devon, England; 3 Department of Agrifood Production and Environmental Sciences (DiSPAA), University of Florence Piazzale delle Cascine, 28 50144 Florence, Italy; 4 The Italian National Research Council – Institute of Biometeorology (CNR-IBIMET), Via Giovanni Caproni, 8 50145 Florence, Italy

Correction to: *Scientific Reports* 10.1038/s41598-018-24459-z, published online 18 April 2018

This Article contains an error in the legend of Figure 1.

“The box signifies the distribution of the 25–75% quartiles, the median is represented by a horizontal line within the box, horizontal bars either side of the box indicate minimum/maximum values. *indicates significant different between the 25 and 35 °C treatments at the 0.05 significance level.”

should read:

“The box signifies the distribution of the 25–75% quartiles, the median is represented by a horizontal line within the box, horizontal bars either side of the box indicate minimum/maximum values. *indicates significant different between the 25 and 35 °C treatments at the 0.05 significance level.”

