# Supplementary material for: Impaired photosynthesis and increased leaf construction costs may induce floral stress during episodes of global warming over macroevolutionary timescales
Source: Sci Rep. 2018 Apr 18;8:6206. doi: 10.1038/s41598-018-24459-z (PMC6049339; doi:10.1038/s41598-018-24459-z)
Supplement: Supplementary file 1 — Supplementary Information [file 41598_2018_24459_MOESM1_ESM.docx]

**Supplementary Information**

**Manuscript Title**: Impaired photosynthesis and increased leaf construction costs may induce floral stress during episodes of global warming over macroevolutionary timescales

**Authors**: Matthew Haworth^1*^, Claire M. Belcher^2^, Dilek Killi^3^, Rebecca A. Dewhirst^2^, Alessandro Materassi^4^, Antonio Raschi^4^ and Mauro Centritto^1^

# ^1^The Italian National Research Council - Tree and Timber Institute (CNR-IVALSA)

^2^University of Exeter

^3^Department of Agrifood Production and Environmental Sciences (DiSPAA), University of Florence

^4^The Italian National Research Council – Institute of Biometeorology (CNR-IBIMET)

**Supplementary Information Figure 1** – the spectrum of light received by the *Ginkgo biloba* plants in the controlled environment chambers. Light sources were Lumilux De Lux 36/965 (Biolux), Power Star HQI-TS 400 W/D and Colour 77 Fluora (Osram SPA, Milan, Italy). For more details of the controlled environment chambers see Materassi et al. (2005: Rivista di Ingegneria Agraria 4:79-87).
